# Widening Consumer Access to Medicines through Switching Medicines to Non-Prescription: A Six Country Comparison

**DOI:** 10.1371/journal.pone.0107726

**Published:** 2014-09-24

**Authors:** Natalie J. Gauld, Fiona S. Kelly, Nahoko Kurosawa, Linda J. M. Bryant, Lynne M. Emmerton, Stephen A. Buetow

**Affiliations:** 1 Department of General Practice and Primary Health Care, University of Auckland, Auckland, New Zealand; 2 School of Pharmacy, University of Auckland, Auckland, New Zealand; 3 Griffith Health Institute, Griffith University, Brisbane, Queensland, Australia; 4 Hokkaido Pharmaceutical University School of Pharmacy, Otaru, Hokkaido, Japan; 5 School of Pharmacy, Faculty of Health Sciences, Curtin University, Perth, Western Australia, Australia; China Agricultural University, China

## Abstract

**Background:**

Switching or reclassifying medicines with established safety profiles from prescription to non-prescription aims to increase timely consumer access to medicines, reduce under-treatment and enhance self-management. However, risks include suboptimal therapy and adverse effects. With a long-standing government policy supporting switching or reclassifying medicines from prescription to non-prescription, the United Kingdom is believed to lead the world in switch, but evidence for this is inconclusive. Interest in switching medicines for certain long-term conditions has arisen in the United Kingdom, United States, and Europe, but such switches have been contentious. The objective of this study was then to provide a comprehensive comparison of progress in switch for medicines across six developed countries: the United States; the United Kingdom; Australia; Japan; the Netherlands; and New Zealand.

**Methods:**

A list of prescription-to-non-prescription medicine switches was systematically compiled. Three measures were used to compare switch activity across the countries: “progressive” switches from 2003 to 2013 (indicating incremental consumer benefit over current non-prescription medicines); “first-in-world” switches from 2003 to 2013; and switch date comparisons for selected medicines.

**Results:**

New Zealand was the most active in progressive switches from 2003 to 2013, with the United Kingdom and Japan not far behind. The United States, Australia and the Netherlands showed the least activity in this period. Few medicines for long-term conditions were switched, even in the United Kingdom and New Zealand where first-in-world switches were most likely. Switch of certain medicines took considerably longer in some countries than others. For example, a consumer in the United Kingdom could self-medicate with a non-sedating antihistamine 19 years earlier than a consumer in the United States.

**Conclusion:**

Proactivity in medicines switching, most notably in New Zealand and the United Kingdom, questions missed opportunities to enhance consumers' self-management in countries such as the United States.

## Introduction

In contrast to her British counterpart, an American woman can now self-medicate for urinary incontinence. Conversely, without a prescription, this American woman cannot access a statin for her moderate cardiovascular risk, unlike in the United Kingdom (UK); nor can she effectively treat her urinary tract infection, unless she is visiting New Zealand (NZ). Such examples of variation in switching (or reclassifying) medicines from prescription to non-prescription availability have implications for consumer access and healthcare. For example, switching cold medications in the 1970s resulted in nearly two million fewer doctor consultations for colds per year in the US [1], and an OTC switch of triptans was estimated to save health funders €75 million in one year across six European countries [2].

In the UK, government policy has long encouraged self-care, including through switching medicines [3]. Such a sustained government interest in medicines switching is uncommon internationally, although occasionally governments have driven switches to enable consumer access [4] or reduce health funding costs [5], [6].

In the early 2000s, stakeholder groups in the UK [7], and Europe [8] identified medicines for long-term conditions and antibiotics as potential switch candidates. While contentious [9], [10], interest in switches outside of the traditional minor ailment arena is continuing. For example, the American College of Obstetricians and Gynecologists recommended a switch of the oral contraceptive to reduce unintended pregnancy [11], and others have proposed this action given the cancer-prevention benefits of combined oral contraceptives [12]. Furthermore, in 2012, the United States (US) Food and Drug Administration (FDA) proposed that switching medicines may address the under-treatment of some chronic conditions [13]. In light of shortages of primary care physicians [14] and escalating healthcare costs in the US [15], switching medicines may indeed reduce some barriers to access.

Concerns about switching include inaccurate diagnosis [16], suboptimal therapy [17], and inappropriate use, including misuse [18]. On the global stage, however, switching is taking place, without reversals, and with support from some governments.

To have an informed perspective on, and be engaged in, change involving medicines switches, the health professionals and policy makers require understanding of what is happening across health systems under mounting pressure to provide better value for money. Switches in one country may provide ideas for widening consumer access to medicines in another country, and an opportunity to learn from another country's experience.

Apart from isolated comparisons of individual medicines [4], [5], international progress in switching medicines, including those for long-term conditions, has not been systematically documented. The US Government Accountability Office (GAO) [19] and Gilbert and colleagues [20] found that countries varied in the number of active ingredients available without prescription, but provided no indication of the nature of the variation, or switch activity over time. Although the UK is believed to lead the world in switch [3], neither of these studies confirmed this belief, perhaps reflecting methodological deficiencies [21]. Thus, this paper aims to assess variation in switching medicines from prescription to non-prescription among selected developed countries.

## Methods

We identified similarly-developed countries ranking highly (45 or above) on education, health, and socioeconomic development and functioning, in the United Nations Human Development Index [22]. Countries were then purposively sampled to represent diversity in medicines schedules, geographical location, population, health system funding, and culture. The countries compared were the US, UK, Japan, the Netherlands, Australia, and NZ. Inclusion of Australia and NZ allowed comparisons between two countries with similar histories, societies, locations, welfare systems, and medicines classifications. Uniquely, these two countries have attempted to harmonize their scheduling since the mid-1990s [21].

The US has a single non-prescription category which does not restrict the medicines to pharmacies or require a pharmacist to be present (open availability) [23]. The UK has two non-prescription classifications: pharmacy-only and general sales (open availability) [23]. Other countries have three non-prescription classifications. NZ and Australia have pharmacist-only, pharmacy-only, and general sales classifications [21]. The Netherlands has pharmacy-only, drugstore and pharmacy, and general sales classifications [23]. Japan restricts non-prescription medicine sales to pharmacists only (category one medicines), or supply by a registered person or pharmacist (category two and category three medicines) [23]. The trained registered person may supply category two or three medicines without a pharmacist present. “Quasi-drugs” such as vitamins and gargles can be sold from stores that do not have a pharmacist or registered person on staff.

The lead author accessed documents from the US, UK, NZ, and Australia that identified prescription-to-non-prescription switches and their dates. US data were derived from the Consumer Healthcare Products Association website [24] and validated against news articles in academic journals. UK sources included regulator consultation documents on switches, *Pharmaceutical Journal* news articles, and industry and pharmaceutical society documents [25], [26]. For Japan, NK and NG ascertained dates of switches from official government websites [27] and other documents [28], [29] as required. For the Netherlands, the pharmaceutical professional organization (Koninklijke Nederlandse Maatschappij ter bevordering der Pharmacie; KNMP) and industry organization (Neprofarm) provided information in the absence of local records in English. NZ and Australian source documents included meeting records for scheduling committees (2000–2013 for Australia and 1990–2013 for NZ), an Australian PhD thesis [30], and the Australian Self-Medication Industry for dates otherwise unavailable.

In this paper, we have used the term ‘switch’ to mean a move from prescription to non-prescription availability whether over-the-counter or provided behind-the-counter (as with pharmacist-only supply in Australia). Switches were analysed on three measures. The first measure compared prescription-to-non-prescription switches from 2003 to 2013, with a specially developed descriptor, “progressive” switches (also known as “innovative” switches). This measure adapted the methodology used for FDA priority reviews for registering new chemical entities, and has been described elsewhere [21]. A progressive switch for an individual country was one providing incremental consumer benefit, either:

(1) safe and effective therapy where no satisfactory non-prescription therapy existed in that country; or

(2) a clinically-significant improvement compared to current non-prescription therapies in that country.

Authors NG, FK, LB, and LE agreed whether or not the medicine met the progressive switch criteria, with input from NK for Japan. Use of these criteria for Australia and NZ has been outlined elsewhere [21]. Table S1 in Supporting Information illustrates the use of these criteria for the other countries. The second measure determined whether a medicine switch was, to the best of our knowledge, the first switch for that type of drug in the developed world (“first-in-world”) from 2003 to 2013. The final measure compared switch dates for pharmacological classes of medicines, or selected medicines in the case of non-steroidal anti-inflammatory agents (to highlight variation between countries) or for medicines unique to a class (e.g. orlistat). These comprised medicines that were progressive switches in the selected countries from 2003 to 2013, along with five switches that occurred across most or all countries before 2003 (ibuprofen, H_2_-antagonists, nicotine replacement therapy, dermal hydrocortisone 1%, and mast cell stabilizers), and an early switch limited to Australia and the US (short-acting beta-agonists).

## Results

**Figure 1:**
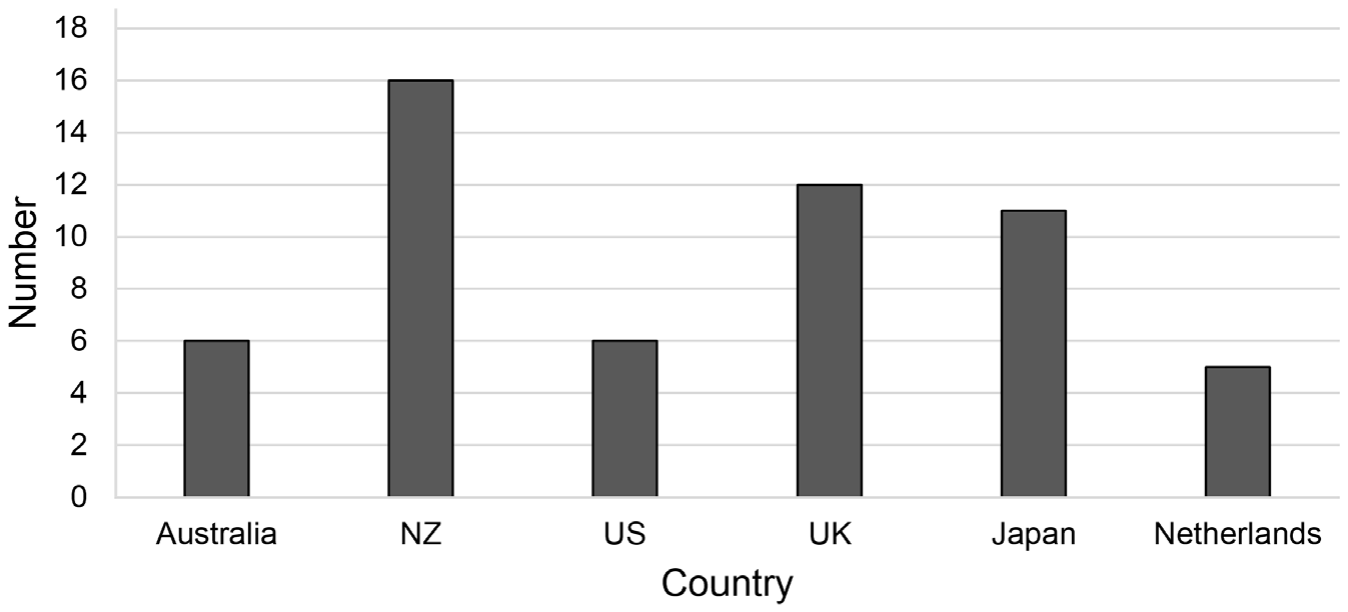
Progressive medicine switches 2003–2013.

Prescription to non-prescription switch activity varied between and within countries over time (Figure 1, Table 1). NZ, the UK, and Japan were the most active during the study period, with 11–16 progressive switches each. In contrast, the US, Netherlands, and Australia had around half of the switches experienced by Japan and the UK. Variation over time in Table 1 includes a recent slowdown in progressive switches in the UK, and inactive periods in the US and Australia. Japan was particularly active in switches in 2007–2008. Much of NZ's recent activity has been driven by vaccine switches, unlike other countries.

**Table 1:** Progressive switches, 2003–2013.

Year	Australia	NZ	US	UK	Japan	Netherlands
2003	EHC; fluconazole		Omeprazole; loratadine^a^	Omeprazole		
2004	Orlistat	Fluconazole; orlistat		Simvastatin; hyoscine (transdermal)	Minoxidil (scalp)^a^	
2005	Pantoprazole	Alclometasone (dermal)		Chloramphenicol (ocular)		EHC
2006		Sumatriptan; oseltamivir	EHC	Sumatriptan; amorolfine (nail)	Triamcinolone (mouth)	
2007			Orlistat		Aciclovir (dermal); flavoxate; tranexamic acid^b^; isoconazole (vaginal)	
2008		Omeprazole		Naproxen; azithromycin	Nicotine (transdermal); minoxidil (scalp)^a^	Omeprazole
2009		Famciclovir; zolmitriptan (nasal); chloramphenicol (ocular)		Orlistat; tamsulosin	Loxoprofen^a^	Ipratropium (nasal); orlistat
2010	Chloramphenicol (ocular)	Calcipotriol (dermal)		Domperidone^a^; tranexamic acid	Beclometasone (nasal)	
2011	Famciclovir	Cholera and ETEC vaccine				Clotrimazole (vaginal)
2012		Influenza vaccine (injection); trimethoprim			Eicosapentaenoic acid (EPA)^c^	
2013		Meningococcal vaccine (injection); tetanus-diphtheria-pertussis vaccine (injection); herpes zoster vaccine (injection)	Oxybutynin (transdermal); triamcinolone (nasal)			

Note: All medicines are oral preparations, unless otherwise stated. ETEC  =  enterotoxigenic *Escherichia coli*; EHC  =  emergency hormonal contraception.

a. Extended indication or increased strength rather than new switch.

b. In combination with ascorbic acid, L- cysteine, pantothenic acid, and pyridoxine, for chloasma.

c. For hypertriglyceridaemia.

The UK and NZ had the most first-in-world switches during 2003–2013 (Table 2). In both countries, these switches included departures from the self-diagnosable minor ailments traditionally considered the domain of non-prescription medicines, e.g. simvastatin, tamsulosin, and topical calcipotriol for long-term use. Most of these medicines have not been subsequently switched in any of the other selected countries.

**Table 2:** First-in-world medicine switches, 2003–2013.

UK	NZ	Australia	Netherlands	US	Japan
Simvastatin	Oseltamivir	Orlistat	Nil	Oxybutynin transdermal	Nil
Sumatriptan	Famciclovir				
Azithromycin	Calcipotriol				
Tamsulosin	Trimethoprim				
	Herpes zoster vaccine				

The final measure, comparing switch dates for selected medicines (Table 3), shows considerable delays in some switches, particularly in the US and Japan compared with others. Japan appears less progressive than its recent activity (Figure 1) would suggest.

**Table 3:** Comparison of timing of selected medicine switches across countries up to and including 2013.

Medicine or class of medicines	UK	NZ	Aust-ralia	Nether-lands	US	Japan
Inhaled short-acting beta agonist	**X**	**X**	1976^a^	**X**	**X** ^b^	**X**
Urinary bladder spasm treatment (flavoxate)	**X**	<1990^c^	<1990^c^	**X**	**X**	2007
Non-sedating antihistamine	1983^d^	<1990	≤1992	≤1995	2002	1990
Ibuprofen	1983	1985	1989	∼1987	1984	1985
Naproxen	2008	≤1990	1983^a^	1996	1994	**X**
Dermal hydrocortisone 1%	1987	1990	∼1997	**X**	1991	**X** ^e^
Nicotine replacement (any form)	1991	<1990	1988^a^	∼1992	1996	2001
Vaginal azole antifungal	1992	1990	1994	2011	1990	2007
Dermal nucleoside analogue (e.g. aciclovir)	1993	1990	1996	≤2000	**X**	2007
Nasal corticosteroid	1994	1996	1999	**X**	2013	2010
H_2_-antagonist	1994	1993	1995	≤1996	1995	1997
Steroid for local oral use	1994	1991	1996	**X**	**X**	2006
Mast cell stabilizer (any form)	1994	1991	<1990	≤1995	1997	1996
Azole antifungal (oral, single dose)	1995	2004	2003	**X**	**X**	**X**
Mebeverine	1997	**X**	**X**	**X**	**X**	**X**
Domperidone	1998	**X**	**X**	<1991	**X**	**X**
Dermal moderate potency corticosteroid	2001	2005	2000	**X**	**X**	**X**
Orlistat	2009	2004	2004	2009	2007	**X**
Proton pump inhibitor	2003	2008	2005	2008	2003	**X**
Emergency hormonal contraceptive	2001	2001	2003	2005	2006	**X**
Statin	2004	**X**	**X**	**X**	**X**	**X**
Ocular chloramphenicol	2005	2009^f^	2010^f^	**X**	**X** ^f^	**X** ^f^
Triptan	2006	2006	**X**	**X**	**X**	**X**
Neuraminidase inhibitor (oseltamivir)	**X**	2006	**X**	**X**	**X**	**X**
Chlamydia treatment (azithromycin)	2008	**X**	**X**	**X**	**X**	**X**
Alpha-1 blocker (tamsulosin)	2009	**X**	**X**	**X**	**X**	**X**
Oral antiviral for herpes labialis	**X**	2009	2011	**X**	**X**	**X**
Tranexamic acid	2010	**X**	**X** ^g^	**X**	**X**	2007^h^
Dermal calcipotriol	**X**	2010	**X**	**X**	**X**	**X**
Cholera and travellers' diarrhoea vaccine	**X**	2011	**X**	**X**	**X**	**X**
Influenza vaccination	**X**	2012	**X**	**X**	**X**	**X**
Trimethoprim	**X**	2012	**X**	**X**	**X**	**X**
Transdermal oxybutynin	**X**	**X** ^i^	**X**	**X**	2013	**X**
Meningococcal vaccine	**X**	2013	**X**	**X**	**X**	**X**
Tetanus-diphtheria-pertussis vaccine	**X**	2013	**X**	**X**	**X**	**X**
Herpes zoster vaccine	**X**	2013	**X**	**X**	**X**	**X**

**X** =  not switched.

a. In early Australian switches timing differed between the States and Territories, the earliest switch date is shown.

b. Orciprenaline (metaproterenol), a non-selective beta-agonist was switched in the US in 1982, but reversed in 1983 [31].

c. Flavoxate is non-prescription in both Australia and NZ, but marketed in neither.

d. The non-sedating antihistamine switched in the UK in 1983 was terfenadine, which later reverted to prescription medicine following QT prolongation concerns [3].

e. Hydrocortisone 1% with oxytetracycline (but not alone) has long been available without prescription in Japan.

f. Other antibacterial eye preparations have long been available without prescription in these jurisdictions, e.g. sulfacetamide in NZ and Australia, polymyxin and bacitracin in the US, and sulfamethoxazole in Japan.

g. Tranexamic acid was switched in Australia in 2000 but never marketed as a non-prescription medicine and reverted to prescription in 2007 under Trans-Tasman Harmonization.

h. Tranexamic acid in Japan was switched at a lower dose, in combination with other ingredients, and for a different indication to the UK (chloasma not menorrhagia) [32].

i. Oral oxybutynin was previously available without prescription in NZ.

Note: Medicines are oral unless otherwise specified. All vaccines are injected except for the cholera and travellers' diarrhoea vaccine which is oral.

Some switches exhibit a “ripple effect”, with widening access in one country which is then followed elsewhere e.g. ibuprofen and H_2_-antagonists (Table 3). Large delays between countries sometimes occurred. US consumers needed a prescription for non-sedating antihistamines, and for nasal corticosteroids for 19 years longer than did UK consumers. Vaginal antifungal switches were considerably delayed in the Netherlands and Japan compared with the US.

While typically the UK had removed the prescription restriction of many medicines earlier than the other countries, naproxen was exceptionally delayed compared with most other countries. The US switched some medicines relatively early, e.g. ibuprofen, vaginal antifungals, and proton pump inhibitors. The early (1982) US switch of the asthma reliever, orciprenaline (metaproterenol), was quickly reversed, unlike the Australian switch of the asthma reliever, salbutamol (albuterol) which still remains switched. Japan differed most from the other countries, in switching medicines that are not marketed in the other countries examined (see Table S1 in Supporting Information), and in different strengths, doses or indications to other countries, e.g. tranexamic acid. Australia and NZ switched many of the same medicines until the mid-2000s then diverged. All three measures of switch showed NZ to be more active than Australia currently, but, unlike Australia, NZ has not switched an inhaled asthma reliever.

## Discussion

While the UK has broken new ground with switches, the effect of government support for switch appears to be waning. NZ was the most progressive in switch of the countries studied, across all measures used. Consumers in the more restrictive US and the Netherlands have continued to need to access doctors for a number of common medicines that have been switched in the UK and NZ. The number of progressive switches differed three-fold across the six selected countries in the 11 years to 2013.

This research demonstrated considerable differences between countries in switching certain identical medicines or classes of medicines. The difference in access may bring advantages and disadvantages to medical care, consumers and society in the different health systems. For example, Americans required a prescription for non-sedating antihistamines for considerably longer than all other countries, despite safety benefits over sedating antihistamines [33], which have long been non-prescription. Sedating antihistamines have been associated with work-place, car, and aviation accidents [34]. Dutch women with vaginal candidiasis required a prescription for vaginal antifungals until 2011, 21 years later than in the US and NZ, with potentially unnecessary doctor workload, higher costs for the health funder, and prolonged discomfort and inconvenience for women. Retaining vaginal antifungals as prescription medicines might have minimized inappropriate use (including unnecessary consumer costs) and encouraged earlier diagnosis of serious conditions. However, American [35] and Finnish [36] doctors supported continued non-prescription availability of vaginal antifungals, despite some doctors reporting consumer self-care deficiencies with vaginal antifungals.

The early (1983) UK switch of the non-sedating antihistamine terfenadine was later reversed following evidence of cardiac effects [3]. While this example suggests that delaying switch might sometimes be beneficial, new switches occurring in the selected countries over the last 10 years have not been reversed on account of safety reasons. Considering the variation in switching medicines with a benign safety profile and limited risk of masking serious conditions, e.g. non-sedating antihistamines and mast cell stabilizers, our data suggest that factors that are not safety-related may delay switch.

Other researchers have reported international variation in medicines availability [19], [20], but by providing only a numerical comparison and including “me-toos” and obsolete drugs [21], the implications for consumer access and medical management are unclear. Our research is the first to show the nature of the international variation through assessing multiple countries over time and using multiple tools.

Opinion seems divided on whether a pharmacy-only or pharmacist-only category affects non-prescription availability [19], [20]. We found that the US, with a single open non-prescription category, appeared less active in switch than the UK, and NZ, which have pharmacist-only and/or pharmacy-only classes. Low switch activity in the Netherlands (despite a pharmacy-only class) and the differences between NZ and Australia (despite similar schedules and attempted harmonization of scheduling) suggest other factors are also involved. In NZ some switched medicines, e.g. vaccines and trimethoprim, can only be supplied under strict criteria and through specially trained pharmacists [21], possibly enabling switches.

We found that few long-term medicines were switched, despite interest in such switches in multiple jurisdictions [7], [8], [13]. The UK has been most active in this field, but most medicines for long-term conditions identified by the UK working party [7] remain prescription-only. Despite statin switch attempts in the US [17] and NZ [21], only the UK had switched a statin. Some may view the reluctance to switch statins positively, given concerns expressed in the UK about efficacy, compliance, and unknown hazards [10]. Although suggested as suitable for non-prescription supply [12], oral contraceptives have not switched in any of the six countries. Our data do not show why such switches have not occurred.

In some countries, mechanisms other than switch may increase consumer access to prescription medicines, as with vaccines in the US [37], and widening prescribing rights to non-physician practitioners [3], possibly circumventing some of the need for switch.

A full investigation of reasons for the variation across countries and over time is warranted, and a subject of our ongoing research. Evaluating the appropriateness, and risks and benefits of each switch would be difficult since little post-switch research is conducted. Dr June Raine from the UK's Medicines and Healthcare products Regulatory Agency (MHRA) believed that for the UK's switches *“…the benefits of wider access to medicines overwhelmingly outweigh the risks”* [38]. Indeed, none of the new switches from the last decade in the selected countries has been reversed, providing confidence in the process. However, spontaneous adverse event reporting has deficiencies [39], and will not identify under-treatment, over-treatment, and delayed diagnosis.

This study used information-rich multi-dimensional means to highlight differences in switch activity between six countries. It moves away from reliance on industry tables [19], [20], and a simple quantitative comparison irrespective of consumer advantage. We focused on the move from prescription-only to non-prescription availability, recognizing the strong potential impact on access and doctor involvement with this change.

We have sought to validate our findings by triangulating switch data with multiple documents or consulting with knowledgeable persons in each country. Official meeting records (as used in NZ and Australia) were not triangulated with other sources. Finding appropriate data and assessing whether or not a switch was progressive were difficult when little information was available in English (e.g. Japan and the Netherlands). Switch dates used may be committee decision dates, official gazettal dates or product launch dates. However, overall findings would not have differed with occasional changes in how the progressive criteria were applied or the slight variation in dates used. Furthermore, comparing all switches would have been less informative than comparing progressive switches because of “me-too” switches in some countries. The finding of diversity in switch may reflect the countries chosen. Had selected countries been more similar, switches might have been more homogeneous, but the variation between NZ and Australia suggests that even similar countries may differ in switch activity.

## Implications

Consumer access to medicines through switch differs across countries with similarly educated consumers. This difference suggests that the health system in some countries could be unnecessarily burdened by managing conditions that may reasonably be self-managed or pharmacist-managed instead. Providing vaccinations through pharmacists, as in NZ, may reduce some barriers to access, and therefore have public health benefits. Perhaps pharmacy or pharmacist-supply availability, as occurs in the more active countries, may help widen consumer access to medicines in the US.

Our findings raise questions as to why countries vary in switching medicines. They also invite investigation into consumer outcomes of the differences in access. These outcomes pertain to realized access *versus* potential access [40], cost, and the safety and quality of healthcare. Widespread problems following the last 10 years of switches have not become evident, nor have recent switches been reversed, but post-marketing surveillance studies of switched medicines to confirm their safety are rare. Outcome data, including multi-country comparisons of outcomes from differences in switch, could be used to explore realized benefits and risks of the differences seen and help to inform further switches.

## Conclusion

Our multi-dimensional study provides benchmarking of switch, and therefore consumer access to medicines, in the six selected countries, and allows other countries to compare their activity with our findings. We found variation between countries in switch activity, and variation in activity over time within some countries. Progressive in this area, the UK and NZ appear willing to approve ground-breaking switches, although the UK may be slowing down. Other countries require of their consumers a prescription for some medicines switched elsewhere, indicating scope for the Netherlands, Australia, and the US to widen access to medicines through switch.

## Supporting Information

Table S1 **Allocation of prescription to non-prescription switches into progressive or non-progressive.**
(DOC)

## References

[1] Temin P (1992) Realized benefits from switching drugs. J Law Econ 35: 351–370.

[2] Millier A, Cohen J, Toumi M (2013) Economic impact of a triptan Rx-to-OTC switch in six EU countries. PLoS ONE 8: e84088. 24367628 10.1371/journal.pone.0084088 PMC3868654

[3] Blenkinsopp A, Bond C (2005) Over the counter medication. London: British Medical Association Board of Science.

[4] Armstrong ME (2006) Plan B contraceptive and the role of politics in medicine: a comparative analysis of the “switch” of emergency contraception from prescription to non-prescription in the United States, France, the United Kingdom, and Canada. The Berkeley Electronic Press (bepress): 1806.

[5] Cohen J (2003) Switching omeprazole in Sweden and the United States. Am J Ther 10: 370–376. 12975722 10.1097/00045391-200309000-00010

[6] Juul P (1991) Prescription-to-OTC: is Denmark a model for the world? Swiss Pharma 13: 100–104.

[7] Proprietary Association of Great Britain. (2002) Potential candidates for reclassification from POM to P. Available: www.pagb.co.uk. Accessed 18 Jan 2011.

[8] World Self-Medication Industries (2009) Switch: prescription to non-prescription switch. Ferney-Voltaire, France.

[9] Dryden MS, Cook J, Davey P (2009) Antibiotic stewardship - more education and regulation not more availability? J Antimicrob Chemother 64: 885–888. 19729376 10.1093/jac/dkp305

[10] Editorial (2004) OTC statins: a bad decision for public health. Lancet 363: 1659. 15158622 10.1016/S0140-6736(04)16284-3

[11] Committee on Gynecologic Practice (2012) Committee opinion no 544: Over-the-counter access to oral contraceptives. Obstet Gynecol 120: 1527–1531. 23168791 10.1097/01.AOG.0000423818.85283.bd

[12] Editorial (2008) The case for preventing ovarian cancer. Lancet 371: 275. 18294977 10.1016/S0140-6736(08)60139-7

[13] Kux L (2012) Using innovative technologies and other conditions of safe use to expand which drug products can be considered nonprescription; public hearing. Fed Regist 77: 12059–12062.

[14] Ghorob A, Bodenheimer T (2012) Sharing the Care to Improve Access to Primary Care. N Engl J Med 366: 1955–1957. 22621625 10.1056/NEJMp1202775

[15] Emanuel EJ (2013) Will physicians lead on controlling health care costs? JAMA 310: 374–375. 23917285 10.1001/jama.2013.60073

[16] Editorial (2005) Over-the-counter triptans–making the switch. Lancet Neurol 4: 587. 10.1016/S1474-4422(05)70174-5 16168921

[17] Tinetti ME (2008) Over-the-counter sales of statins and other drugs for asymptomatic conditions. N Engl J Med 358: 2728–2732. 18565867 10.1056/NEJMsb0801202

[18] Roussin A, Bouyssi A, Pouché L, Pourcel L, Lapeyre-Mestre M (2013) Misuse and dependence on non-prescription codeine analgesics or sedative H1 antihistamines by adults: a cross-sectional investigation in France. PLoS ONE 8: e76499. 24098516 10.1371/journal.pone.0076499 PMC3789666

[19] United States Government Accountability Office (2009) Nonprescription drugs. Considerations regarding a behind-the-counter drug class.

[20] Gilbert A, Rao D, Quintrell N (2006) A review of pharmaceutical scheduling processes in six countries and the effect on consumer access to medicines. Int J Pharm Pract 14: 95–104.

[21] Gauld N, Kelly F, Emmerton L, Bryant L, Buetow S (2012) Innovations from ‘down-under’: a focus on prescription to non-prescription medicine reclassification in New Zealand and Australia. SelfCare 3: 88–107.

[22] United Nations Development Programme (2010) Human Development Report. 20th ed. New York: Ed Palgrave McMillan.

[23] AESGP (2011) Economic and Legal Framework for Non-Prescription Medicines. Brussels: AESGP.

[24] Consumer Healthcare Products Association (2013) Ingredients & dosages transferred from Rx-to-OTC status (or new OTC approvals) by the Food and Drug Administration since 1975. Washington DC, USA. Available from: http://www.chpa-info.org/media/resources/r_4620.pdf Accessed: 19 Nov 2013.

[25] Royal Pharmaceutical Society of Great Britain (2008) RPS e-PIC References on: Prescription only medicines reclassified to pharmacy only medicines. London.

[26] Proprietary Association of Great Britain (PAGB) (2012) POM - P Switches. London.

[27] Pharmaceutical and Medical Devices Agency (2012) [Information of approved assessment for OTC medicine]. Available from: http://www.info.pmda.go.jp/approvalSrch/OverTheCounterSrchInit? Accessed 12 Nov 2012.

[28] Maekawa H, Saito K (2012) [New movement of switch OTC medicines]. Iyaku J 48: 883–888.

[29] Sasaki K, Tomioka M (2003) [Pharmacy newsletter for pharmacy student no. 8]. Available from: http://www10.showa-u.ac.jp/~sucenter/ynews/aynews8.pdf. Accessed 12 Nov 2012.

[30] Bowden M (1993) Schedule 3 medicines and the prescription to non-prescription switch: a survey of community pharmacists. Sydney: University of Sydney.

[31] Hendeles L, Weinberger M (1984) Nonprescription sale of inhaled metaproterenol–deja vu. N Engl J Med 310: 207–208. 6690937 10.1056/NEJM198401193100326

[32] Pharmaceuticals and Medical Devices Agency (PMDA) (2007) [Examination report for approval].

[33] Kay GG (2000) The effects of antihistamines on cognition and performance. J Allergy Clin Immunol 105: S622–S627. 10856168 10.1067/mai.2000.106153

[34] Canfield DV, Dubowski KM, Chaturvedi AK, Whinnery JE (2012) Drugs and alcohol found in civil aviation accident pilot fatalities from 2004–2008. Aviat Space Environ Med 83: 764–770. 22872990 10.3357/asem.3306.2012

[35] Taylor CA, Lipsky MS (1994) Physicians' perceptions of the impact of the reclassification of vaginal antifungal agents. J Fam Pract 38: 157–160. 8308507

[36] Sihvo S, Hemminki E, Ahonen R (1999) Physicians' Attitudes Toward Reclassifying Drugs as Over-the-Counter. Med Care 37: 518–525. 10335754 10.1097/00005650-199905000-00011

[37] American Pharmacists Association (2012) Pharmacist Administered Vaccines: Types of vaccines authorized to administer. Available from: http://www.pharmacist.com/AM/Template.cfm?Section=Pharmacist_Immunization_Center1&ContentID=26931&Template=/CM/ContentDisplay.cfm. Accessed on 11 Jul 2013.

[38] Raine J (2008) Updated:- Reclassification and OTC medicines under continual review (rapid response). BMJ: 10 Apr 2008.

[39] Clark D, Layton D, Shakir SA (2001) Monitoring the safety of over the counter drugs. We need a better way than spontaneous reports. BMJ 323: 706–707. 11576964 10.1136/bmj.323.7315.706 PMC1121271

[40] Andersen RM (1995) Revisiting the behavioral model and access to medical care: does it matter? J Health Soc Behav 36: 1–10. 7738325

