# Supplementary material for: Widening Consumer Access to Medicines through Switching Medicines to Non-Prescription: A Six Country Comparison
Source: PLoS One. 2014 Sep 24;9(9):e107726. doi: 10.1371/journal.pone.0107726 (PMC4175460; doi:10.1371/journal.pone.0107726)
Supplement: Table S1 — Allocation of prescription to non-prescription switches into progressive or non-progressive. (DOC) [file pone.0107726.s001.doc]

**Table S1 Allocation of prescription to non-prescription switches into progressive or non-progressive**

| **Medicine** | **Year** | **Progressive?** | **Reasoning** |
| --- | --- | --- | --- |
| **United Kingdom** |  |  |  |
| Omeprazole | 2003 | Yes | More effective than H_2_ antagonists [1] |
| Hyoscine (transdermal) | 2004 | Yes | One patch lasts 3 days rather than dosing 2-3 times daily |
| Simvastatin | 2004 | Yes | First statin to switch |
| Chloramphenicol (eye drops) | 2005 | Yes | First antibacterial eye drop |
| Alclometasone diproprionate (dermal) | 2005 | No | Clobetasone butyrate previously switched |
| Amorolfine (nail lacquer) | 2006 | Yes | Low efficacy [1], but no other nail antifungals OTC [2,3] |
| Sumatriptan | 2006 | Yes | Triptans used in migraines non-responsive to simple analgesics [1] |
| Penciclovir (dermal) | 2006 | No | Aciclovir previously switched |
| Naproxen | 2008 | Yes | Maximum daily dose 750 mg [2]. Longer acting and higher relative dosing (to prescription) compared with ibuprofen [1]. |
| Azithromycin | 2008 | Yes | First non-prescription antibacterial for Chlamydia |
| Diclofenac | 2008 | No | Maximum daily dose 75 mg [2] similar relative dosing (to prescription) compared with non-prescription ibuprofen [1]. |
| Tamsulosin | 2009 | Yes | First licensed medicine switched for benign prostatic hypertrophy |
| Pantoprazole | 2009 | No | Omeprazole already switched |
| Orlistat | 2009 | Yes | First licensed medicine for weight loss switched |
| Tranexamic acid | 2010 | Yes | No previous non-prescription treatment for menorrhagia |
| Domperidone | 2010 | Yes | Extended indication. No previous non-prescription nausea and vomiting treatment (except for in migraine) [2]. |
| Diclofenac (patch) | 2011 | No | The patch (for local pain) unlikely to have substantially greater benefit than existing non-prescription oral and topical anti-inflammatories |
| Ibuprofen (dermal) | 2012 | No | For sunburn. Other non-prescription sunburn products are available |
| Rabeprazole | 2012 | No | Omeprazole switched previously |
| Esomeprazole | 2013 | No | Omeprazole switched previously |
|  |  |  |  |
| **United States** |  |  |  |
| Omeprazole | 2003 | Yes | More effective than H_2_ antagonists [1]. |
| Loratadine | 2003 | Yes | Extended indication. First non-sedating antihistamine for hives |
| Ecamsule (dermal) | 2006 | No | Previous sunscreens available |
| Emergency hormonal contraceptive | 2006 | Yes | First emergency contraception switched |
| Terbinafine (dermal) | 2006 | No | Previous dermal antifungals |
| Ketotifen (ocular) | 2006 | No | Other topical antihistamines or mast cell stabilisers available |
| Polyethylene glycol 2250 | 2006 | No | Other laxatives available |
| Orlistat | 2007 | Yes | First such licensed weight loss remedy |
| Cetirizine | 2007 | No | Loratadine already switched |
| Lansoprazole | 2009 | No | Omeprazole already switched |
| Fexofenadine | 2011 | No | Loratadine already switched |
| Oxybutynin (transdermal) | 2013 | Yes |  |
| Triamcinolone (nasal) | 2013 | Yes |  |
|  |  |  |  |
| **Japan** |  |  |  |
| Ketotifen (nasal) | 2004 | No | Sodium cromoglycate previously switched [4] |
| Roxatidine | 2004 | No | H_2_-antagonist previously switched [4] |
| Nizatidine | 2004 | No | H_2_-antagonist previously switched [4] |
| Minoxidil (scalp) | 2004 | Yes | Extension of indication to women |
| Azelastine | 2005 | No | Other oral non-sedating antihistamine already available [4] |
| Ketotifen | 2006 | No | Other oral antihistamines already available [4] |
| Tiquizium bromide | 2006 | No | Scopolamine already switched for stomach cramps in 1987 [4] |
| Lanoconazole (dermal) | 2006 | No | Other topical antifungals already available [4] |
| Triamcinolone (mouth) | 2006 | Yes |  |
| Aciclovir (dermal) | 2007 | Yes |  |
| Ketotifen (ocular) | 2007 | No | Sodium cromoglycate previously switched [4] |
| Ambroxol | 2007 | No | Bromhexine already non-prescription [5] |
| Flavoxate | 2007 | Yes | First medicine for urinary incontinence switched |
| Tranexamic acid | 2007 | Yes | Unique medicine for liver spots |
| Isoconazole (vaginal) | 2007 | Yes | First vaginal antifungal switched |
| Miconazole (vaginal) | 2008 | No | Isoconazole already switched |
| Nicotine (transdermal) | 2008 | Yes | Gum switched [4], but patch provides even nicotine levels and is not addictive [6] |
| Emedastine | 2008 | No | Other oral antihistamines |
| Minoxidil (five times strength) | 2008 | Yes | Stronger than previous formulation |
| Diclofenac (dermal) | 2009 | No | Topical anti-inflammatories previously switched |
| Vidarabine (dermal) | 2009 | No | Other antivirals already switched |
| Troxipide (combination) | 2009 | No | Teprenone previously switched for gastritis [4] |
| Epinastine | 2009 | No | Similar to azelastine |
| Loxoprofen | 2009 | Yes | Non-prescription dose same as prescription dose. Oral ibuprofen previously switched, but in lower dose than prescription [4,7,8,9] |
| Clotrimazole (vaginal) | 2010 | No | Other vaginal antifungals already switched |
| Oxyconazole (vaginal) | 2010 | No | Other vaginal antifungals already switched |
| Beclometasone (nasal) | 2010 | Yes | First nasal corticosteroid |
| Oxymetazoline (nasal) | 2010 | No | Other vasoconstrictors already available [5] |
| Acitazanolast (ocular) | 2011 | No | Leukotriene inhibitor but no evidence of improvement over alternatives^b^ |
| Pemirolast | 2011 | No | Similar to ketotifen [1] |
| Mequitazine | 2011 | No | Increased dose; others non-prescription have comparable dosing |
| Ibuprofen | 2012 | No | Increased dose, but loxoprofen already available at same as prescription dose |
| Fexofenadine | 2012 | No | Other antihistamines available |
| Neticonazole vaginal | 2012 | No | Other vaginal antifungals switched |
| Eicosapentaenoic acid | 2012 | Yes | This appears to be the first switch for hypertriglyceridaemia |
| Cetirizine hydrochloride | 2012 | No |  |
| Tranilast (eye drops) | 2013 | No | Other allergic conjunctivitis preparations already available |
| Trimebutine | 2013 | No | Other antispasmodics already available |
| Ebastine | 2013 | No | Other antihistamines already available |
|  |  |  |  |
| **The Netherlands** |  |  |  |
| Terbinafine (topical) | 2004 | No | Other topical antifungals previously switched |
| Ambroxol (lozenge) | 2004 | No | Other throat lozenges previously switched with similar properties |
| EHC | 2005 | Yes |  |
| Docosanol (dermal) | 2008 | No | Other dermal antivirals for herpes labialis switched |
| Fexofenadine | 2008 | No | Other non-prescription non-sedating antihistamines |
| Omeprazole | 2008 | Yes | First proton pump inhibitor |
| Ipratropium (nasal) | 2009 | Yes | First nasal anticholinergic |
| Orlistat | 2009 | Yes |  |
| Pantoprazole | 2009 | No | Omeprazole already switched |
| Flurbiprofen (lozenge) | 2010 | No | Benzydamine lozenges switched previously |
| Clotrimazole (vaginal) | 2011 | Yes | First vaginal antifungal |

Medicines listed are oral unless otherwise stated.

Combination medicines and nutritionals excluded. EHC = emergency hormonal contraception

a. As loxoprofen non-prescription is available at prescription strength, it has been deemed innovative. It is unknown if prescription dosing for ibuprofen and loxoprofen are equivalent. No head-to-head studies in humans arose from a Medline search (12 Nov 2012). b. Acitazanolast has little information in English; topical leukotriene inhibitors do not appear in allergic conjunctivitis in Martindale [1], or in Medline indexing.

1. Sweetman SC, editor. (2010) Martindale: The Complete Drug Reference. London: The Pharmaceutical Press.

2. MHRA List C Consolidated list of substances which are present in authorised products which have been reclassified since 1 April 2002. Available from: <http://www.mhra.gov.uk/Howweregulate/Medicines/Licensingofmedicines/Legalstatusandreclassification/Listsofsubstances/index.htm> Accessed: 17 Jan 2014

3. Ryan RSM, editor. (2012) British National Formulary. London: BMJ Group Pharmaceutical Press.

4. Sasaki K, Tomioka M (2003) [Pharmacy newsletter for pharmacy student no. 8].

5. Ministry of Health, Labour and Welfare. (2002) [List of switch OTC in main countries: Japan, US, UK, Germany, France]. Available from: <http://www.mhlw.go.jp/shingi/2002/11/dl/s1108-4e.pdf> Accessed: 12 Nov 2012

6. Shaw JP, Ferry DG, Pethica D, Brenner D, Tucker IG (1998) Usage patterns of transdermal nicotine when purchased as a non-prescription medicine from pharmacies. Tob Control 7: 161-167.

7. Daiichi Sankyo Healthcare Limited [Loxonin-s package insert]. Daiichi Sankyo.

8. Taiyo Pharmaceuticals (2012) [Ibuprofen 200mg datasheet]. Japan Pharmaceutical Information Centre.

9. Pharmaceutical and Medical Devices Agency (2012) [Information of approved assessment for OTC medicine]. Available from: <http://www.info.pmda.go.jp/approvalSrch/OverTheCounterSrchInit>? Accessed 12 Nov 2012
